# Incidence of acute kidney injury in critically burned patients resuscitated with crystalloid and colloid according to parameters of transpulmonary thermodilution, diuresis and lactic acid

**DOI:** 10.1186/cc14438

**Published:** 2015-03-16

**Authors:** P Extremera Navas, M Sanchez Sanchez, I Pozuelo Echegaray, A Agrifoglio Rotaeche, A Robles Caballero, A García de Lorenzo

**Affiliations:** 1 Hospital Universitario la Paz, Madrid, Spain

## Introduction

The purpose was to study the incidence of acute kidney injury (AKI) according to RIFLE and AKIN criteria in critically ill burn patients resuscitated with Ringer's solution and supplements of lower molecular weight hydroxyethyl starch (HES)130/0.4/6%, and to determine the relationship between RRT indication and mortality.

## Methods

We studied 165 consecutive patients admitted to the critical care burn unit. Resuscitation was performed using lactated Ringer's solution and HES at a low dose to achieve urine output, lactate levels, and transpulmonary thermodilution parameters. The contributions of colloids and crystalloids were measured, and renal function was evaluated. Statistical analysis was performed using the Spearman test.

## Results

The average total body surface area (TBSA) burned was 30 ± 15%, and the median of the total volume needed in the first 24 hours was 4.01 ml/kg/% TBSA burned. According to the RIFLE criteria, 10 (6.1%) patients presented with risk, 11 (6.7%) presented with injury, and 11 (6.7%) presented with failure. According to the AKIN criteria: 9.7% presented stage I, 3% stage II and 10.3% stage III. Replacement therapy (RRT) was performed in 15 patients (9.1%). In six of these patients RRT was employed in the final stages of multiorgan failure. In the remaining nine patients, for various reasons only one survived.

## Conclusion

During the resuscitation phase of the burn patients, the use of HES (130/0.4/6%) at low doses does not seem to cause more risk or injury according to RIFLE or AKIN criteria than those reported by studies in burn patients resuscitated without HES. However, the need for RRT is associated with a high mortality, although in many cases the display is terminal.

